# Desethylamiodarone—A metabolite of amiodarone—Induces apoptosis on T24 human bladder cancer cells via multiple pathways

**DOI:** 10.1371/journal.pone.0189470

**Published:** 2017-12-08

**Authors:** Zita Bognar, Katalin Fekete, Csenge Antus, Eniko Hocsak, Rita Bognar, Antal Tapodi, Arpad Boronkai, Nelli Farkas, Ferenc Gallyas, Balazs Sumegi, Arpad Szanto

**Affiliations:** 1 Department of Biochemistry and Medical Chemistry, University of Pecs, Pecs, Hungary; 2 Department of Oncotherapy, University of Pecs, Pecs, Hungary; 3 Institute of Bioanalysis, University of Pecs, Pecs, Hungary; 4 MTA-PTE Nuclear-Mitochondrial Research Group, Pecs, Hungary; 5 Szentagothai Research Center, University of Pecs, Pecs, Hungary; 6 Department of Urology, University of Pecs, Pecs, Hungary; Duke University School of Medicine, UNITED STATES

## Abstract

Bladder cancer (BC) is a common malignancy of the urinary tract that has a higher frequency in men than in women. Cytostatic resistance and metastasis formation are significant risk factors in BC therapy; therefore, there is great interest in overcoming drug resistance and in initiating research for novel chemotherapeutic approaches. Here, we suggest that desethylamiodarone (DEA)–a metabolite of amiodarone—may have cytostatic potential. DEA activates the collapse of mitochondrial membrane potential (detected by JC-1 fluorescence), and induces cell death in T24 human transitional-cell bladder carcinoma cell line at physiologically achievable concentrations. DEA induces cell cycle arrest in the G0/G1 phase, which may contribute to the inhibition of cell proliferation, and shifts the Bax/Bcl-2 ratio to initiate apoptosis, induce AIF nuclear translocation, and activate PARP-1 cleavage and caspase-3 activation. The major cytoprotective kinases—ERK and Akt—are inhibited by DEA, which may contribute to its cell death-inducing effects. DEA also inhibits the expression of B-cell-specific Moloney murine leukemia virus integration site 1 (BMI1) and reduces colony formation of T24 bladder carcinoma cells, indicating its possible inhibitory effect on metastatic potential. These data show that DEA is a novel anti-cancer candidate of multiple cell death-inducing effects and metastatic potential. Our findings recommend further evaluation of its effects in clinical studies.

## Introduction

Bladder cancer is the most significant malignancy of the urinary tract worldwide and accounts for about 3% of all cancer-related deaths. It is considerably more frequent in men than in women [1,2]. Urothelial cell carcinoma, the most common pathologic subtype of bladder cancer, is observed in over 90% of tumors [3,4]. Fortunately, about 80% of patients with nonmuscle invasive cancer can be successfully treated using surgery. Approximately 20–30% of bladder cancer patients present with an aggressive tumor that invades the muscle, and more than half of these patients develop distant metastases [5]. Patients with invasive bladder cancer require a radical cystectomy. After surgery chemo-, radio- and immunotherapy can be used to improve survival, but the prognosis of invasive bladder cancer still remains unsatisfactory. Despite a number of randomized controlled trials, to date there are no data to confirm what the best combination of treatments to treat invasive bladder cancer is [6]. The modest results with current drugs suggest an urgent need to identify new agents [7] that will improve the prognosis of invasive bladder cancer.

**Fig 1:**
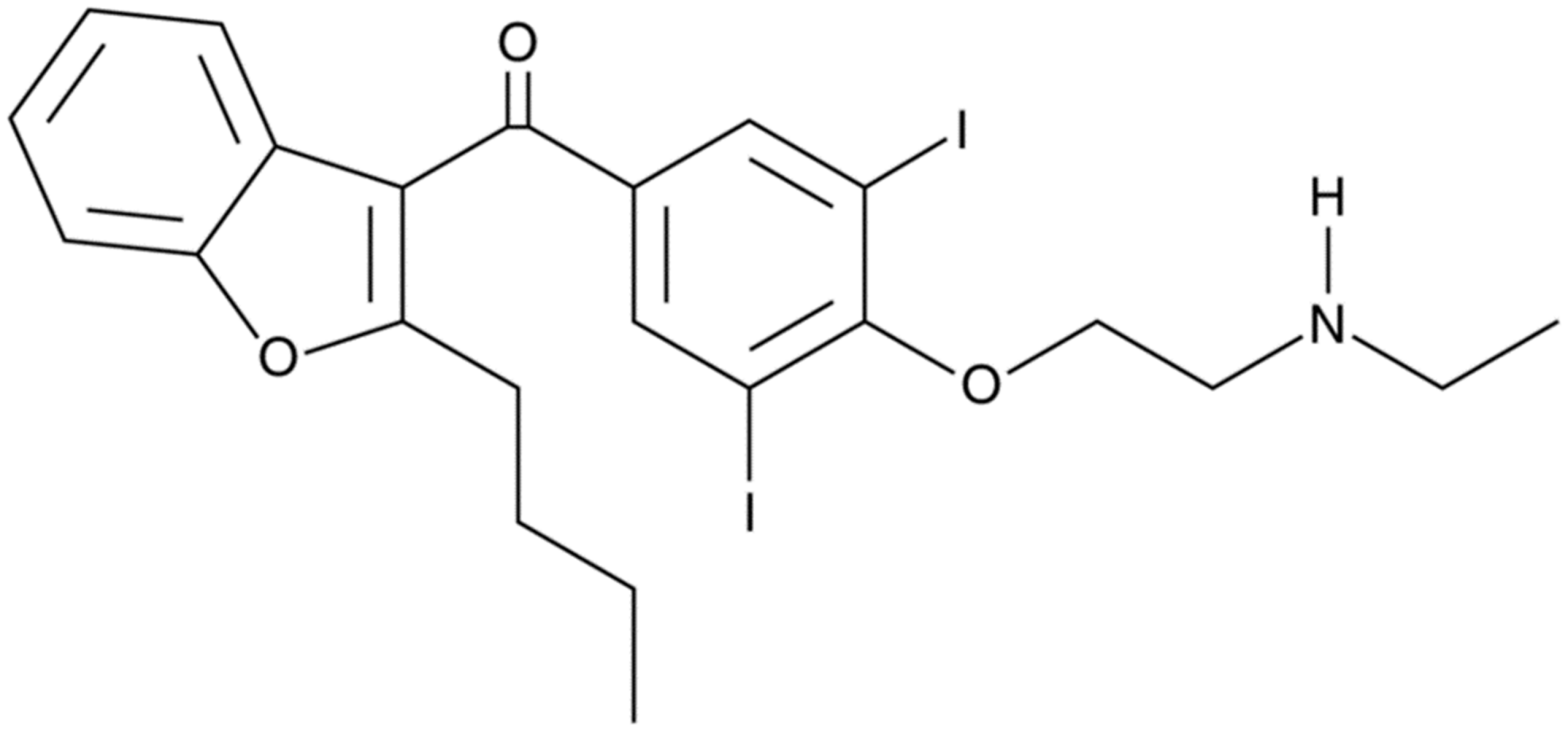
Structure of desethylamiodarone.

Desethylamiodarone (DEA) (Fig 1), the major metabolite of the widely used antiarrhythmic drug amiodarone, is produced in an N-demethylation reaction catalyzed by cytochrome P450 3A4 [8,9]. DEA is also a pharmacologically active compound. It also has antiarrhythmic activity, significantly increasing the action potential duration (class III antiarrhythmic effect) and decreasing the maximum rate of depolarization (class I antiarrhythmic effect) at clinically relevant concentrations [10,11]. After amiodarone treatment, amiodarone and DEA rapidly and extensively accumulate in extracardiac tissues (notably in the liver, lung and adipose tissue), even achieving μmol/g concentrations [12–14] and has a very long elimination half-life [13,15,16]. Tissue concentrations of amiodarone and DEA are 100 times higher than the corresponding plasma concentrations [15,16]. Extensive tissue accumulation of DEA and its long elimination time can give a possible role to DEA in progressive, muscle-invasive bladder cancer treatment.

Disturbed cell cycle control and apoptosis can result in uncontrolled cell proliferation during cancer development [17]. Consequently, the inhibition of apoptosis and the arrest of the cell cycle can be an effective treatment for eliminating cancer. Previous studies in our laboratory indicated that DEA has negative effects on the stability of the mitochondrial membrane system [18]; therefore, we raise the possibility that DEA may have a cytostatic effect on tumor cells at physiologically relevant concentrations.

## Materials and methods

### Cell culture

T24 human bladder carcinoma cells were purchased from the American Type Culture Collection (Wesel, Germany). Cells were maintained in McCoy’s 5A with high glucose, L-glutamine, Bacto Peptone, HEPES and phenol red indicator (Life Technologies, Darmstadt, Germany). Cell medium was supplemented with 10% fetal bovine serum and an antibiotic solution (1% penicillin and streptomycin mixture) (Life Technologies, Darmstadt, Germany). Cells were maintained in a humidified environment at 37°C with 5% CO_2_. They were subcultured twice weekly for up to a maximum of 10 weeks.

### Cell viability assays

For determination of cell viability T24 cells (3 × 10^5^/ml) were plated in 24-well plates, cultured overnight and treated with the indicated concentration of DEA for 24 or 48 hours. The DEA was a kind gift from Professor Dr. András Varró (Department of Pharmacology and Pharmacotherapy, University of Szeged, Szeged, Hungary). Cell viability after DEA treatment was quantified using the Muse^™^ Cell Count & Viability Assay, and the flow cytometry-based Muse^™^ Cell Analyzer (EMD Millipore Bioscience, Darmstadt, Germany) according to the instructions provided by the manufacturer. Cell viability was expressed as the relative percentage of living cells of the untreated control samples. The experiments were performed in quadruplicate and repeated three times.

### Colony formation assay

Cells were trypsinized and plated in triplicate in 6-well plates at a density of 500 cells/well, before being treated with different concentrations of DEA. After 7 days of incubation, the cells were washed and stained with crystal violet, and the colonies containing more than 50 cells were counted. The number of colonies was determined and normalized to the number of colonies in the controls.

### Detection of apoptosis

For the quantitative analysis of apoptosis, we used the Muse^™^ Annexin V & Dead Cell Assay on a Muse^™^ Cell Analyzer. The assay utilizes Annexin V to detect phosphatidylserine on the external membrane of apoptotic cells. Here, 1 × 10^6^ cells were seeded onto regular plates and treated for 6 hours without or with the indicated concentrations of DEA; cells were collected and sample preparations were made. Then, 100 μL of cells in suspension were added to each tube for incubation with 100 μL of the Muse^™^ Annexin V & Dead Cell reagent. After 20 minutes of incubation at room temperature in the dark, the samples were analyzed according to the manufacturer’s protocol.

### Sub-cellular fractionation

Twenty-four hours after thawing, T24 cells, grown and harvested from regular plates, were washed twice with phosphate-buffered saline (PBS) and resuspended in 1 ml fractionation buffer (250 mM sucrose, 20 mM HEPES, pH 7.4, 10 mM KCl, 1.5 mM MgCl_2_, 1 mM EDTA, 1 mM EGTA, 1 mM dithiothreitol (DTT) and proteinase inhibitor cocktail (Sigma, #P2714)). The cell lysate was passed through a Potter homogenizer and incubated on ice for 20 minutes, followed by a 7-minute 720 x g centrifugation at 4°C. The nuclear pellet was rewashed with 700 μl fractionation buffer and homogenized with a Potter homogenizer. After a second 10-minute centrifugation at 500 x g, the pellet was resuspended with 700 μl fractionation buffer, and again homogenized. The solution was centrifuged (600 x g, 4°C, 10 minutes) and the pellet was resuspended in lysis buffer (10% glycerol, 25 mM NaCl, 50 mM NaF, 10 mM Na-pyrophosphate, 2 nM EGTA, 2 nM DTT, 20 nM p-nitrophenyl-phosphate, 25 mM Tris-HCl, pH 7.4, 50 nM beta-glycerolphosphate and 0.1% Triton X-100) to collect the nuclear fraction.

### Detection of DNA fragmentation

Cell apoptosis was assessed using Hoechst 33342 staining (Molecular Probes, Carlsbad, CA, USA). Briefly, replicate cultures of T24 cells were plated in 6-well plates. The cells were treated with or without DEA for 24 hours. After a change of fresh medium, the cells were incubated with Hoechst 33342 solution at 37°C for 10 minutes, followed by examination under a fluorescence microscope. Strong fluorescence and condensed or fragmented nuclei were observed in apoptotic cells, while weak fluorescence was observed in live cells. The quantification of apoptotic cells was performed by taking images in random fields and counting at least 200 cells in four random fields per well. The nuclear DNA in the treated cells contained in 6-well plates was visualized by staining with the DNA-specific dye Hoechst 33342 at a final concentration of 5 μg/ml. The cells were observed immediately with filters for blue fluorescence.

### Detection of mitochondrial membrane potential (Δψ)

The changes in Δψ were assayed using JC-1 dye, which is taken up by the mitochondria. T24 cells were seeded at 1 × 10^6^ cells/well in a 6-well plate containing coverslips and cultured at least overnight before the experiment. After subjecting the cells to different concentrations of DEA, the coverslips were rinsed twice in PBS and then placed upside down on top of a small chamber formed by a microscope slide filled with PBS supplemented with 0.5% fetal calf serum and containing 5 μg/ml JC-1 dye (Molecular Probes). The cells were imaged with a Nikon Eclipse Ti-U fluorescent microscope (Auro-Science Consulting Ltd., Budapest, Hungary), which was equipped with a Spot RT3 camera, using a 60x objective lens with epifluorescent illumination. For JC-1 fluorescence, the cells were loaded with the dye for 15 minutes, and then the same microscopic field was imaged first with 546 nm bandpass excitation and 590 nm emission (red), then with green filters. Under these conditions, we did not observe considerable bleed-through between the red and green images.

### Cell cycle assay

After treatment with DEA (10 μM) for 24 hours, cells were collected by centrifugation at 211 x g for 5 minutes, washed with ice-cold PBS, fixed with 70% ethanol, stained with a premixed reagent, which included the nuclear DNA intercalating stain propidium iodide (PI) and RNAse A in a proprietary formulation, and analyzed according to the manufacturer’s protocol. PI discriminates cells at different stages of the cell cycle, based on differential DNA content in the presence of RNAse to increase the specificity of DNA staining. The Muse Cell Cycle Software Module performs calculations automatically.

### Immunoblot analysis

Here, 1 × 10^6^ cells were seeded onto regular plates and treated for the cell viability assay. Cells were harvested at intervals in a chilled lysis buffer containing 0.5 mM sodium-metavanadate, 1 mM EDTA and protease inhibitor cocktail (1:200), all purchased from Sigma—Aldrich Co. (Budapest, Hungary). Cell lysates were boiled and subjected to 10% sodium dodecyl sulfate polyacrylamide gel electrophoresis before being transferred to nitrocellulose membranes. The membranes were blocked in 5% low-fat milk for 1.5 hours at room temperature before being exposed to primary antibodies at 4°C overnight in a blocking solution. The following antibodies were used: polyclonal caspase-3 (clone H-277), polyclonal poly(ADP-ribose) polymerase 1 (PARP-1), polyclonal B-cell-specific Moloney murine leukemia virus integration site 1 (BMI1), polyclonal phospho-extracellular signal-regulated kinase (ERK1/2) (Thr202/Tyr204), polyclonal phospho-GSK-3β (Ser9), polyclonal phospho-Akt (Ser473), polyclonal Bcl-2, polyclonal Bax (each 1:500 dilution), monoclonal histone H1 (HH1; 1:200), monoclonal apoptosis-inducing factor (AIF) (1:200) and monoclonal glyceraldehyde-3-phosphate dehydrogenase (GAPDH) (1:2000, clone 6C5). Antibodies were purchased from Cell Signaling Technology (Beverly, MA, USA) except caspase 3, PARP-1, BMI1, HH1 and AIF, which were bought from Santa Cruz Biotechnology (Wembley, UK), while GAPDH antibody was obtained from EMD Millipore Bioscience. Appropriate horseradish peroxidase-conjugated secondary antibodies were used at a dilution of 1:5000 (anti-mouse and anti-rabbit IgGs; Sigma—Aldrich Co.) and visualized by enhanced chemiluminescence (Amersham Biosciences, Piscataway, New Jersey, USA). The films were scanned, and the pixel volumes of the bands were determined using NIH Image J software (Bethesda, Maryland, USA). For membrane stripping and reprobing, the membranes were washed in a stripping buffer (0.1 M glycine, 5 M MgCl_2_, pH 2.8) for an hour at room temperature. After washing and blocking, the membranes were incubated with primary antibodies for nonphosphorylated or loading control proteins. All experiments were repeated three times.

### Data analysis

All data are expressed as mean ± standard deviation (SD). The concentration-dependent effects of DEA in each experiment were tested with ANOVA using the post hoc Dunnett test. In the case of cell cycle analysis, we used the Mann-Whitney U test to compare the treated example to the untreated control. Differences were considered significant at values of p < 0.05 or lower. Statistical analyses were performed using IBM SPSS Statistics v20.0 (IBM Corporation, New York, USA).

## Results

### Effect of DEA on mitochondrial depolarization

**Fig 2:**
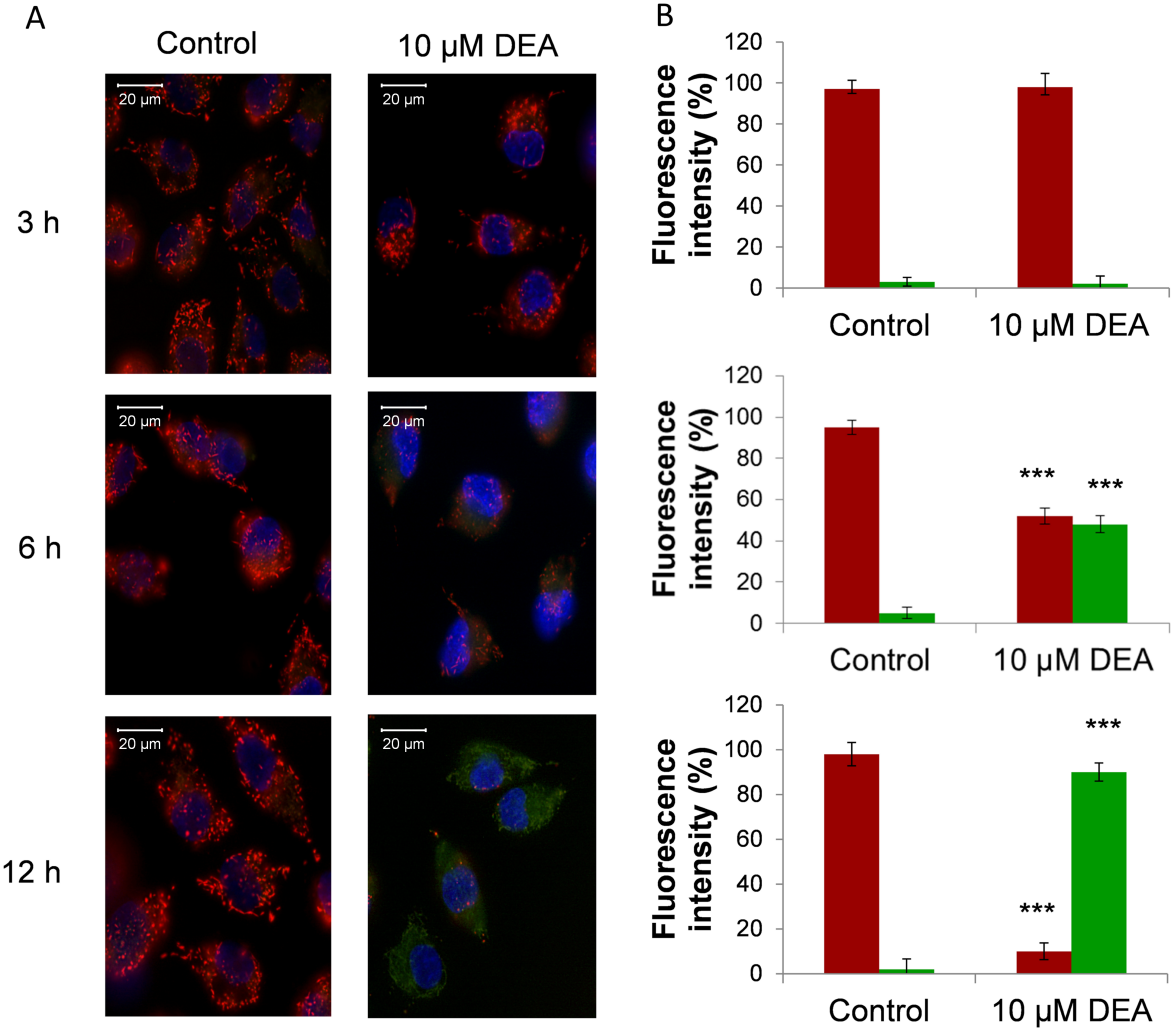
Effect of DEA on mitochondrial depolarization in T24 cells. T24 cells were treated with 10 μM DEA for 3, 6 and 12 hours to induce mitochondrial membrane depolarization. After treatment, the medium was replaced with fresh medium without any agents and containing 1 μM JC-1 membrane potential-sensitive fluorescent dye. After 15 minutes of loading, green and red fluorescence images of the same field were acquired using a fluorescent microscope equipped with a digital camera. (A) The images were merged to demonstrate depolarization of Δψ *in vivo*, indicated by a loss of the red component of the merged image. (B) Representative merged images of three independent experiments and fluorescent intensity in bar diagrams are presented. Data are presented as the mean ± SD. ***p < 0.001 compared to control.

Our previous data showed that DEA at high concentrations destabilized the mitochondrial membrane system in isolated rat liver mitochondria [18]. This observation indicates that DEA may have similar effects on the mitochondria of cancer cells. T24 were treated with 10 μM DEA for 3, 6 and 12 hours to induce mitochondrial membrane depolarization. We analyzed the effect of DEA on mitochondrial depolarization by using JC-1 membrane potential-sensitive dye with fluorescent microscopy. High Δψ is characterized by red (590 nm) fluorescence, while low Δψ is characterized by green (530 nm) fluorescence. DEA treatment (10 μM) induces a time-dependent depolarization of mitochondria in T24 cells while no significant changes in Δψ are observed in the control cells (Fig 2A and 2B). These data show that DEA induces a significant loss of Δψ, which may facilitate the induction of cell death in T24 cells.

### Effects of DEA on cell viability

**Fig 3:**
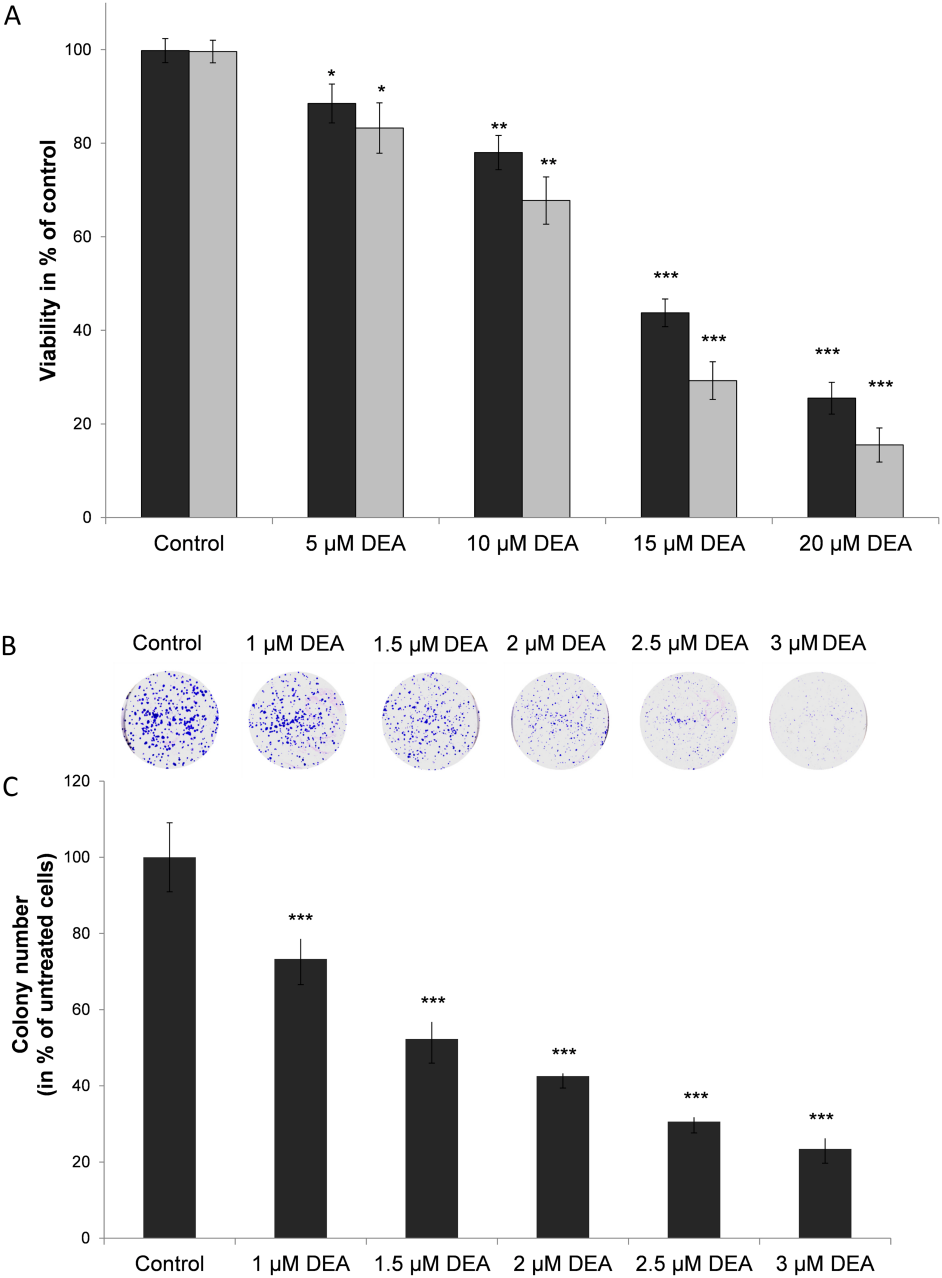
Effect of DEA on cell viability and on colony formation of T24 cells. (A) T24 cells were exposed to increasing concentrations of DEA for 24 (dark grey bars) and 48 hours (light gray bars). Untreated cells served as controls. Data represent means ± SD of three independent experiments performed in at least quadruplicate: *p < 0.05, **p < 0.01 and ***p < 0.001 compared to the corresponding control group. (B) For the colony formation assay T24 cells were exposed to increasing concentrations of DEA for 7 days. The results are presented as representative images of the colony formation assay. The colony-forming abilities are also presented in bar diagrams (C). Untreated cells served as controls. The results are mean ± SD of three independent experiments performed in at least quadruplicate: *p < 0.05, **p < 0.01 and ***p < 0.001 compared to the control group.

T24 cells were treated with increasing concentrations of DEA for 24 and 48 hours. DEA has a statistically significant cell death-inducing effect on T24 cells determined by using the Muse^™^ Cell Count & Viability Assay. The cell death-inducing effect of DEA was more pronounced in the 48-hour study (Fig 3A). These data indicate that DEA induces cell death; therefore, the possible pathways contributing to DEA-induced cell death are analyzed below.

### Effect of DEA on colony formation

The effect of DEA on the colony formation of T24 cells was studied as described above. In our experiment DEA significantly inhibited colony formation in T24 cells even at the lowest concentration (1 μM) we used (Fig 3B and 3C). These data show that DEA can induce cell death, and can inhibit colony formation at low micromolar concentrations, as previously detected *in vivo* in amiodarone-treated animals [10,11,14].

### Effect of DEA on activation of apoptosis in T24 cells

**Fig 4:**
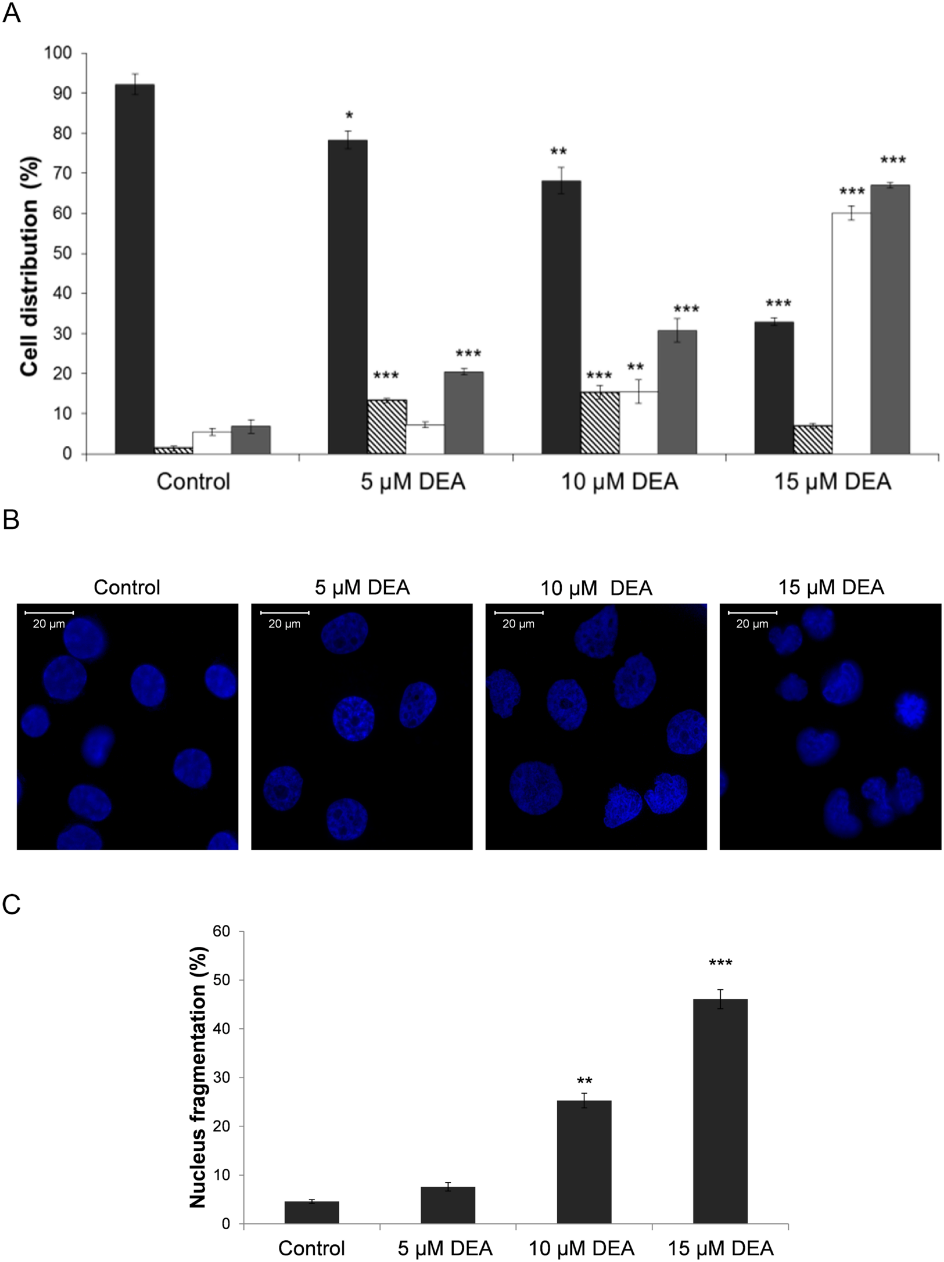
Effect of DEA on activation of apoptosis in T24 cells. T24 cells were treated with increasing concentrations of DEA for 24 hours to induce apoptosis, then stained with the Muse^™^ Annexin V & Dead Cell Reagent, and acquired on the Muse^™^ Cell Analyzer. (A) Graphs demonstrate the percentage of living (dark gray bars), early apoptotic (striped bars), late apoptotic (white bars) and total apoptotic cells (light gray bars). Untreated cells served as controls. The results are mean ± SD of three independent experiments performed in at least quadruplicate: *p < 0.05, **p < 0.01 and ***p < 0.001 compared to the corresponding control group. In order to investigate the fragmentation of nuclei in T24 cells we treated them with increasing concentrations of DEA for 24 hours. Apoptosis was assessed by Hoechst 33342 staining. (B) Quantification of apoptotic cells was performed by taking the images in random fields and counting cells with strong fluorescence, and condensed or fragmented nuclei. (C) The rate of nuclear fragmentation is also presented in bar diagrams. Each column represents the average obtained from three independent experiments. Data are presented as the mean ± SD, **p < 0.01, ***p < 0.001 compared to control.

Flow cytometry with the Muse^™^ Annexin V & Dead Cell Assay was used to investigate the mode of cell death. The assay includes cell surface Annexin V binding, which measured the appearance of phosphatidylserine on the external plasma membrane, a marker of apoptosis. Our results show that DEA increases the total apoptosis rate in a dose-dependent manner (Fig 4A). Increasing DEA concentration shifted the cells from the early to the late apoptosis phase, and only a very low percentage of cells die by necrosis (Fig 4A) T24 cells were treated with increasing concentrations of DEA to study the fragmentation of nuclei stained by Hoechst 33342 (Fig 4B and 4C). These data show that DEA increases the nuclear fragmentation in the 5 to 15 μM concentration range, giving additional support for DEA-induced apoptosis in T24 tumor cells.

### Effect of DEA on Bcl-2-related protein expressions and caspase-3 activation

**Fig 5:**
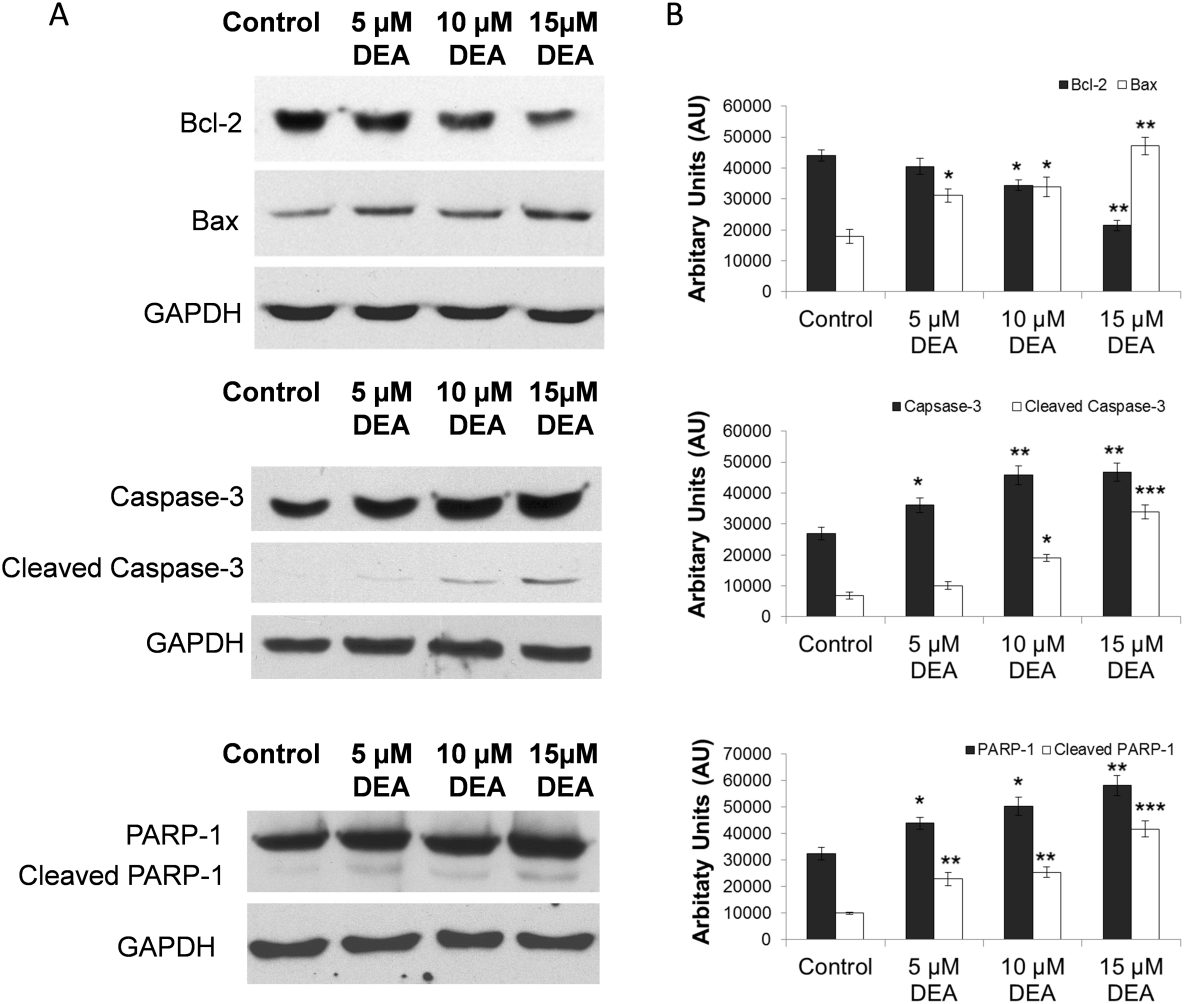
The effect of DEA on the expression of major apoptosis regulatory proteins in T24 cells. T24 cells were exposed to increasing concentrations of DEA for 24-hour intervals. Equal amounts of lysate protein were subjected to gel electrophoresis. Expression levels of Bax, Bcl-2, caspase-3 and PARP were monitored by immunoblot assay. GAPDH was used as loading control. The results are presented as representative immunoblots (A) and densitometric analysis of immunoblots in bar diagrams (B). The results are mean ± SD of three independent experiments: *p < 0.05, **p < 0.01 and ***p < 0.001 compared to the corresponding control group.

In order to understand the potential mechanism of DEA-induced cell death, we studied the effect of DEA on the expression of pro- and anti-apoptotic proteins. DEA treatment decreased the expression of Bcl-2 in a concentration-dependent way, and activated the expression of Bax (Fig 5A and 5B). These data indicate that DEA suppresses the expression of the anti-apoptotic Bcl-2 and increases the expression of the pro-apoptotic Bax; therefore, DEA shifts T24 cells in the direction of apoptosis.

We also analyzed caspase-3 activation, which is best known for its role in the execution of apoptosis, and we found that DEA activates caspase-3 in a concentration-dependent way; moreover, a slight activation in the expression of pro-caspase-3 was also detected (Fig 5A and 5B).

### Effect of DEA on PARP-1 expression and cleavage

Furthermore, DEA was found to enhance dose-dependently the expression of PARP-1 (Fig 5A and 5B), another enzyme that plays a major role in the execution of apoptosis. The increase in expression was also accompanied by cleavage of PARP-1, thereby suggesting the role of active caspase-3 in monitoring the activity of PARP-1 (Fig 5A and 5B). These data indicate that DEA predominantly activates apoptotic cell death through several pathways, which can be an advantage in cancer therapy, where it is possible that one or two pathways can be mutated during the development of cytostatic resistance.

### Effect of DEA on BMI1 protein expression

**Fig 6:**
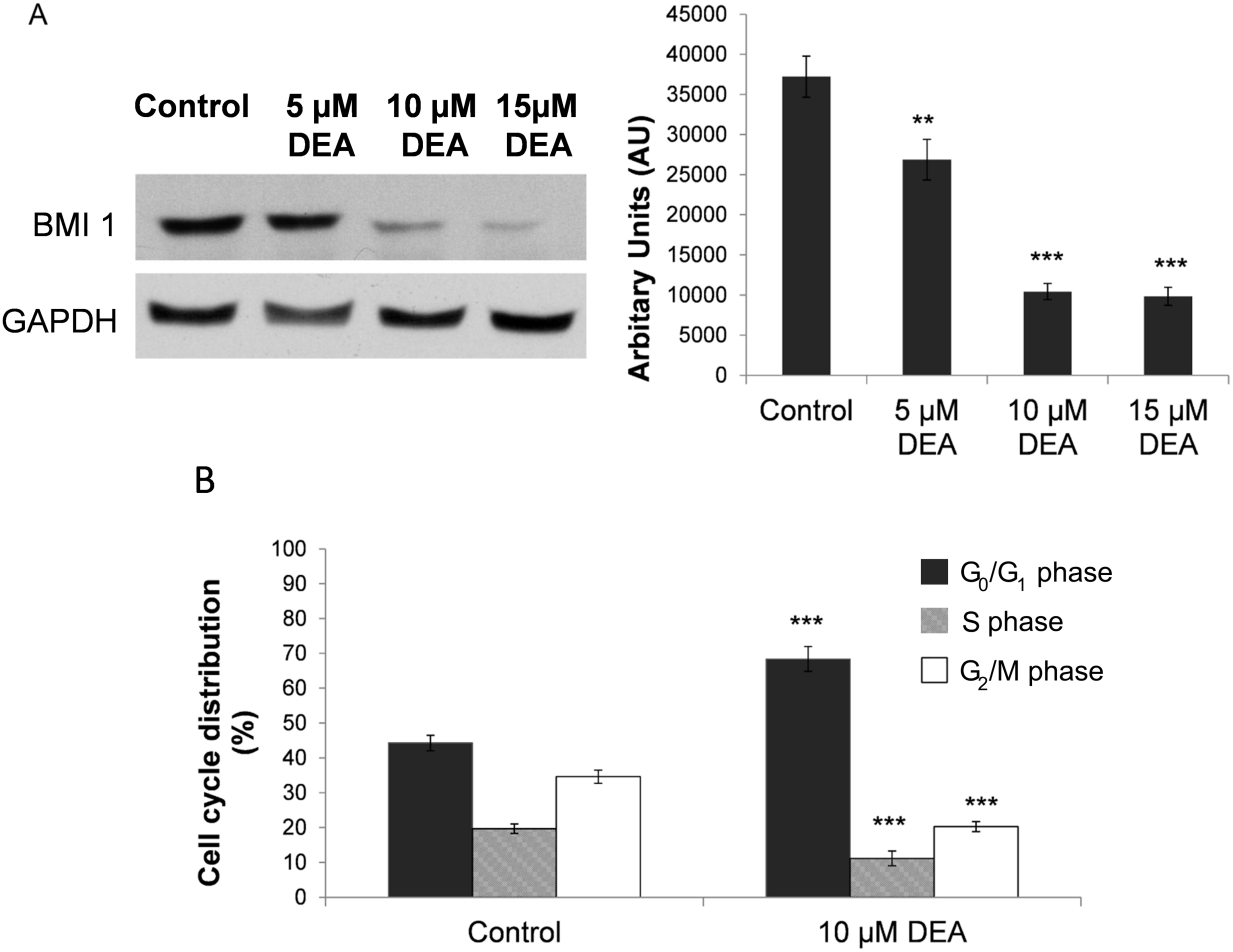
The effect of DEA on the expression of BMI1 and on the cell cycle in T24 cells. (A) T24 cells were exposed to increasing concentrations of DEA for 24-hour intervals. Equal amounts of lysate protein were subjected to gel electrophoresis. Expression levels of BMI1 were monitored by immunoblot assay. GAPDH was used as loading control. The results are presented as representative immunoblots and their densitometric analysis in bar diagram. Data represent mean ± SD of three independent experiments: **p < 0.01 and ***p < 0.001 compared to the corresponding control group. (B) T24 cells were treated with 10 μM of DEA for 24 hours. Cells were harvested, fixed with ethanol and stained with propidium iodide. DNA content was determined using the Muse^™^ Cell Analyzer. Graphs demonstrate the percentage of G0/G1phase (dark gray bars), S phase (striped bars) and G2/M phase (white bars). Untreated cells served as controls. Each column represents the average obtained from three independent experiments. Data are presented as the mean ± SD, ***p < 0.001 compared to control.

Novel studies are suggesting that BMI1 is involved in the proliferation, senescence and migration of cancer and self-renewal of cancer stem cells (CSCs). BMI1 was highly expressed in the T24 bladder cancer cell line and it has been shown to be associated with a very poor prognosis in bladder cancer patients. We analyzed the effect of DEA on the expression of BMI1 and found a concentration-dependent decrease (Fig 6A).

### Effect of DEA on cell cycle arrest

The effect of DEA on the cell cycle in T24 cells was studied at 10 μM DEA for 24 hours. Fig 6B shows that the DEA treatment increases the percentage of cells in the G0/G1 phase from 46.27 ± 2.22% to 65.37 ± 3.58%. At the same time, the percentage of cells in the S phase decreased from 17.66 ± 1.33% to 13.15 ± 2.11%; in addition, the percentage of cells in the G2/M phase was also decreased in the control from 34.59 ± 1.89% to 20.27 ± 1.44% for DEA treatment (Fig 6B). Our data indicate that DEA induces cell cycle arrest in the G0/G1 phase, which can contribute to its cell death-inducing effect.

### Effect of DEA on the AIF translocation to the nucleus

**Fig 7:**
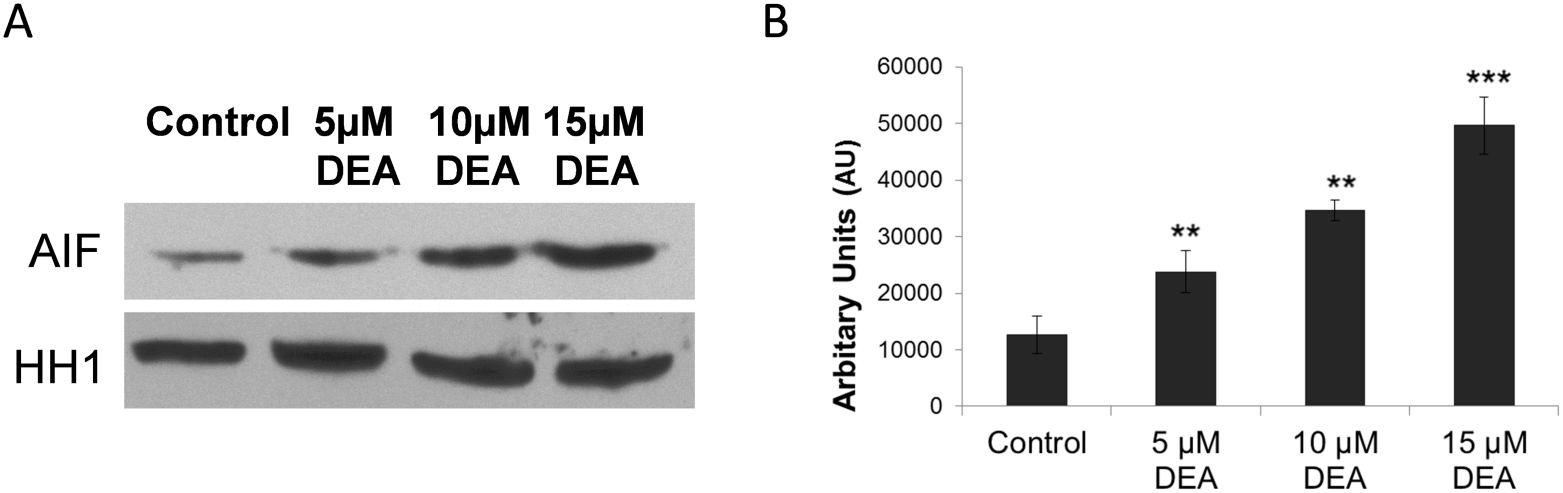
Effect of DEA on the AIF translocation to the nucleus. In order to investigate the Ca^2+^-dependent nuclear translocation of AIF, we treated T24 cells with increasing concentrations of DEA for 24 hours, then we isolated the nuclei. Equal amounts of nuclear fraction protein lysates were subjected to gel electrophoresis. The expression level of AIF was monitored by immunoblot assay. HH1 was used as loading control. The results are presented as representative immunoblots (A) and densitometric analysis of immunoblots in bar diagrams (B). The results are mean ± SD of three independent experiments: **p < 0.01 and ***p < 0.001 compared to the corresponding control group.

DEA was demonstrated to increase cytosolic free Ca^2+^ concentration [19,20], which, in turn, can trigger apoptosis, by upregulating among other mechanisms AIF translocation to the nucleus [21]. Accordingly, we treated T24 cells with increasing concentrations of DEA for 24 hours, isolated the nuclei and assessed AIF nuclear translocation by immunoblotting from the nuclear fraction (Fig 7). Our results show that DEA increases AIF nuclear translocation in a dose-dependent manner.

### Effect of DEA on key signaling pathways

**Fig 8:**
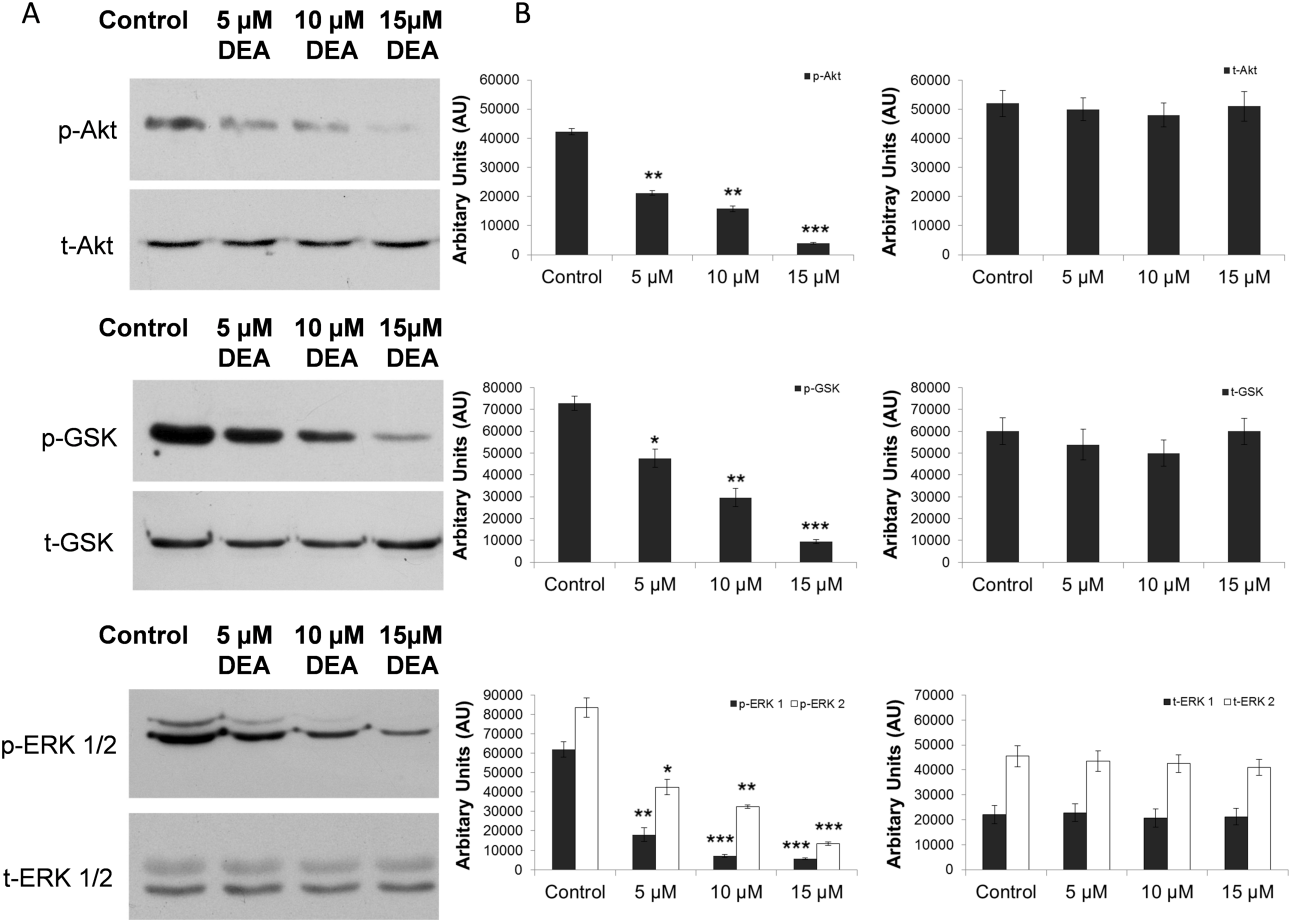
Effect of DEA on Akt, GSK-3β and ERK pathways in T24 cells. T24 cells were treated with increasing concentrations of DEA for 6 hours as indicated. Total cell extracts were analyzed by immunoblotting utilizing anti-total ERK1/2 (ERK), anti-p-ERK1/2 (pERK), anti-Akt (t-Akt), anti-phospho-Akt (p-Akt), anti-GSK (GSK) and anti-phospho-GSK (p-GSK) primary antibodies. The results are presented as representative immunoblots (A) and densitometric analysis of immunoblots in bar diagrams (B). The results are mean ± SD of three independent experiments: *p < 0.05, **p < 0.01 and ***p < 0.001 compared to the corresponding control group.

To contribute to a better understanding of the pro-apoptotic property of DEA, we analyzed cytoprotective kinase pathways. Phosphorylation of Akt (protein kinase B) showed that DEA decreases Akt phosphorylation and activation, which is shown by decreased phosphorylation of its downstream target GSK-3β (Fig 8A and 8B). ERK1/2 is predominantly a cytoprotective kinase and its phosphorylation is also reduced as a consequence of DEA treatment (Fig 8A and 8B). Thus, DEA inactivates Akt and ERK kinases in a concentration-dependent way, without affecting their total protein levels; moreover, this effect of DEA can contribute to the activation of DEA-induced cell death pathways in T24 cells.

## Discussion

In spite of regular chemotherapy followed by radical cystectomy, the tumor cells frequently recur and metastasis may develop [5,7,22]. Consequently, a novel therapeutic strategy is urgently needed to improve the prognosis of patients with bladder cancer. In the current study, we report the ability of DEA (Fig 1), the main metabolite of the antiarrhythmic drug amiodarone, to inhibit growth and induce apoptosis in T24 cells. We found that DEA has significant anti-proliferative and pro-apoptotic effects on T24 cells *in vitro*, which indicates its potential as an antitumor agent for the treatment of bladder cancer (Figs 3 and 4).

Overexpression of the oncogene BMI1 in bladder cancer [23,24], and CSCs [25–27], as well as in various other human cancers has been associated with aggressive tumor behavior and poor outcome. According to the results of previous studies, expression levels of BMI1 in invasive bladder cancer were significantly higher than those detected in superficial bladder cancer, thereby showing a strong correlation with the clinicopathological features of this cancer type. Modulation of the PI3K/Akt pathway has been shown to play a role in cancer cell progression induced by BMI1 [28]. Cell cycle dysregulation due to mutations in one or more tumor-suppressor genes like p53, p14, p16 and retinoblastoma promotes tumorigenesis [29–32]. G1 phase transition during the cell cycle, governed by D-type cyclins, cyclin E, CDK4/6, CDK inhibitors including p16 and p14, and retinoblastoma protein [33–35], is the most effective target of antitumor agents. BMI1 is involved in the chemoresistance of tumor cells through its downstream target ink-14-arf that encodes the tumor suppressor proteins p16 and p14 [28,36].

DEA induced a decrease of BMI1 expression followed by a G0/G1 phase accumulation (Fig 6) accompanied by cell death and increased AnnexinV/PI incorporation, suggesting that G0/G1 phase arrest is related to cell death. Because the p53 gene is mutated in T24 cells [37,38], it is likely that the induction of G1 phase arrest is mediated through p21, in a p53-independent fashion [37]. According to our results, treatment with DEA was able to promote the inhibition of cell proliferation by G1/G0 cell cycle arrest (Fig 6). There is a lot of evidence indicating that G1/G0 arrest occurs in the early steps of apoptosis in different cancer cells following different treatments [39,40].

The balance between apoptosis and cell proliferation determines the rate of tumor progression [17]. Chemotherapy may induce cell cycle arrest and cellular apoptosis in tumor cells, thereby reducing the tumor.

Ras genes have several mutations that can be detected in the process of tumor formation [41,42]. A transitional cell carcinoma of the human urinary bladder predominantly contains H-ras mutations [41]. Signaling pathways leading to ERK are frequently hyperactivated in several tumor cells, which are frequently the consequence of Ras mutation [43]. The ras oncogenic protein overexpressed in T24 cell lines is a common upstream activator of the Raf/MEK/ERK and PI3K/Akt signaling pathways [44–47].

DEA was demonstrated to increase intracellular Ca^2+^ both in the absence and presence of extracellular Ca^2+^, indicating that the [Ca^2+^]_i_ had originated from extracellular, endoplasmic reticular and mitochondrial pools [20]. Additionally, increased [Ca^2+^]_i_ was reported to activate various pro-apoptotic signaling pathways [21]. In complete agreement with these results, we found that DEA induced nuclear translocation of AIF in a concentration-dependent manner (Fig 7). However because of the complex mechanism of DEA’s effect on [Ca^2+^]_i_, we could not determine whether increased [Ca^2+^]_i_ caused or merely accompanied DEA-induced AIF nuclear translocation. For the same reason, it was hard to see whether or not DEA’s effect on various kinase signaling systems involved its effect on [Ca^2+^]_i_. Compounds targeting ERK signaling, like Raf or MEK inhibitors, lead to significant improvements in different tumor types [43,48]. In many cases, initial response rates decrease with the progression of the disease, and the inhibitors somehow become ineffective, inhibiting the ERK pathway and inducing cell death [48]. Therefore, finding new molecules that inhibit the ERK pathway through different mechanisms can involve novel innovative approaches to targeting the ERK pathway. Since the ERK signaling pathway is known to play a critical role in cellular proliferation, we determined whether DEA inhibits cell growth via the regulation of the ERK pathway in T24 cells. DEA significantly inhibited ERK phosphorylation (Fig 8). As ERK is a major mitogenic signal, inactivation of the ERK pathway by DEA appears to be accountable for its anti-proliferative effect. By inhibiting the ERK pathway, DEA can provide a new way to scope ERK overactivated tumors, and can lead to a new tumor therapy against tumor resistance and metastasis formation.

The PI3K/Akt pathway is also known to be important in cell proliferation and survival [49] and the activity of Akt is often altered in human malignancies [50]. This pathway, like the ERK cascade, contributes to the regulation of many proteins involved in apoptosis [47]. In our study, we observed a significant decrease in the phosphorylation of Akt (Fig 8).

Akt triggers the phosphorylation/inactivation of GSK-3β Ser9, which inhibits the degradation of GSK-3β target β-catenin [51], leading to increased nuclear translocation of β-catenin and stimulation of cell proliferation and survival [52]. We therefore determined whether DEA-induced decreased Akt phosphorylation leads to changes in GSK-3β phosphorylation in T24 cells. Indeed, we found that decreased Akt phosphorylation after treatment with 10–15 μM DEA was paralleled by decreased GSK-3β phosphorylation at Ser9 (Fig 8).

It has been shown that ERK and Akt activation contributes to survivin expression and to metastatic progression; therefore, inhibition of both the ERK and Akt pathways would reduce cytostatic resistance and metastatic progression [53]. DEA, by inhibiting both kinases (Fig 8), inducing apoptosis (Figs 4 and 5) and reducing colony formation (Fig 3), can be a potential candidate for preventing the formation of metastasis and can overcome cytostatic resistance. It has been shown that inhibition of the ERK and Akt pathways leads to blocking autophagy and activates cell death [54].

**Fig 9:**
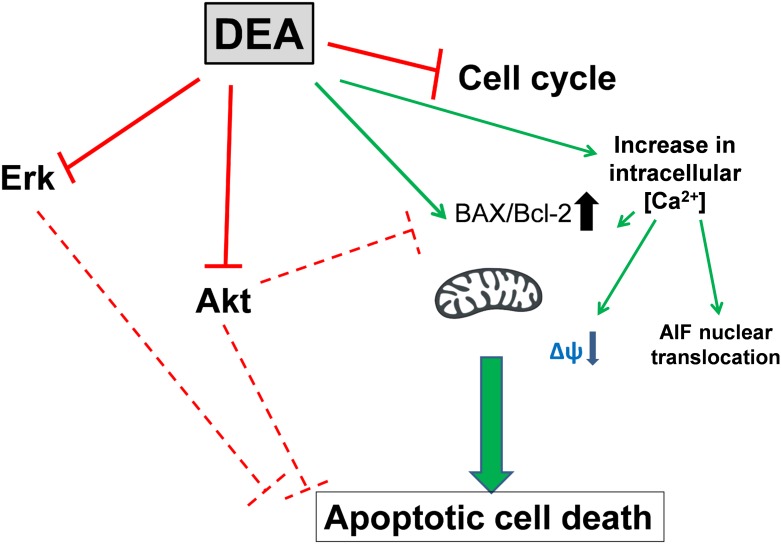
DEA induces tumor cell apoptosis through multiple pathways.

In conclusion, DEA induces tumor cell apoptosis through multiple pathways, including cell cycle arrest, AIF nuclear translocation, PARP-1 cleavage, activation of the Bax/Bcl-2 ratio, caspase-3 activation, and inhibition of the ERK and Akt cytoprotective pathways (Fig 9). This wide range of pathways contributes to its cell death-inducing effects, indicating that DEA can be applied in tumor therapy in cytostatic-resistant tumor cells when apoptotic pathways are mutated. These data show that DEA is a novel anti-cancer agent, with multiple cell death-inducing effects and metastatic potential. Our findings recommend further evaluation of its effects in clinics.
